# Crystal structures of the Schiff base derivatives (*E*)-*N*′-[(1*H*-indol-3-yl)methyl­idene]isonicotino­hydrazide ethanol monosolvate and (*E*)-*N*-methyl-2-[1-(2-oxo-2*H*-chromen-3-yl)ethyl­idene]hydrazinecarbo­thio­amide

**DOI:** 10.1107/S205698901700411X

**Published:** 2017-03-24

**Authors:** Sivaraj Saranya, Jebiti Haribabu, Nattamai S. P. Bhuvanesh, Ramasamy Karvembu, Dasararaju Gayathri

**Affiliations:** a Department of Biotechnology, Dr. M.G.R. Educational and Research Institute University, Maduravoyal, Chennai 600 095, India; b Department of Chemistry, National Institute of Technology, Tiruchirappalli 620 015, India; c Department of Chemistry, Texas A & M University, College Station, TX 77842, USA; d Centre of Advanced Study in Crystallography and Biophysics, University of Madras, Guindy Campus, Chennai 600 025, India

**Keywords:** crystal structure, Schiff base derivatives, isonicotinohydrazide, hydrazinecarbo­thio­amide

## Abstract

The crystal structures of two title Schiff base derivatives, C_15_H_12_N_4_O·C_2_H_6_O (**1·EtOH**) and C_13_H_13_N_3_O_2_S (**2**), were determined at 110 and 100 K, respectively. In the crystal of compound **1·EtOH**, the (*E*)-*N*′-[(1*H*-indol-3-yl)methyl­idene]isonicotinohydrazide and ethanol mol­ecules are linked by O—H⋯O, N—H⋯O and N—H⋯N hydrogen bonds, forming a tape structure running along the *b-*axis direction. The tapes are weakly linked *via* a C—H⋯N inter­action. In the crystal of compound **2**, (*E*)-*N*-methyl-2-[1-(2-oxo-2*H*-chromen-3-yl)ethyl­idene]hydrazinecarbo­thio­amide mol­ecules are linked *via* N—H⋯O and C—H⋯O hydrogen bonds, forming a helical chain along the *b*-axis direction. The chains are further linked into a layer expanding parallel to (102) through C—H⋯S inter­actions.

## Abstract 2

In the two title Schiff base derivatives, the (*E*)-*N*′-[(1*H*-indol-3-yl)methyl­idene]isonicotinohydrazide mol­ecules and (*E*)-*N*-methyl-2-[1-(2-oxo-2*H*-chromen-3-yl)ethyl­idene]hydrazinecarbo­thio­amide mol­ecules form a tape structure and a helical chain structure, respectively, through hydrogen bonds.

## Chemical context   

Schiff base derivatives are a biologically versatile class of compounds possessing diverse activities, such as anti-oxidant (Haribabu, Subhashree *et al.*, 2015 ▸, 2016 ▸), anti-inflammatory (Alam *et al.*, 2012 ▸), anti-cancer (Creaven *et al.*, 2010 ▸; Haribabu, Jeyalakshmi *et al.*, 2015 ▸, 2016 ▸), anti-bacterial (Sondhi *et al.*, 2006 ▸), anti-fungal (Jarrahpour *et al.*, 2007 ▸), anti-convulsant (Bhat & Al-Omar, 2011 ▸). Schiff bases have gained special attention in pharmacophore research and in the development of several bioactive lead mol­ecules. They are widely used as catalysts, corrosion inhibitors and inter­mediates in organic synthesis, and also play a potential role in the development of coordination chemistry (Muralisankar *et al.*, 2016 ▸). As part of our studies in this area, we have synthesized the title Schiff base compounds, **1·EtOH** and **2**, and determined their crystal structures.

## Structural commentary   

**Figure 1:**
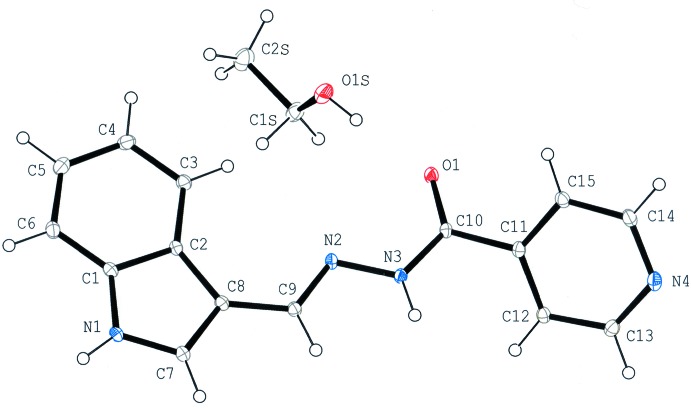
The mol­ecular structure of compound **1·EtOH**, with the atom labelling. Displacement ellipsoids of non-H atoms are drawn at 30% probability level.

The mol­ecular structures ((Figs. 1 ▸ and 2 ▸) of both **1** and **2** are non-planar, as evidenced by the torsion angles N3—C10—C11—C12 [42.5 (3)°] in **1** and C1—C2—C10—N1 [−152.0 (2)°] in **2**. The mean plane of the central chain C9/N2/N3/C10/O1 in **1** makes dihedral angles of 6.91 (12) and 42.71 (13)°, respectively, with the C1–C8/N1 ring system and the pyridine C11–C15/N4 ring. In mol­ecule **2**, the dihedral angle between the C1–C9/O1 ring system and the mean plane of the C10/N1/N2/C12/N3/C13 chain is 30.36 (9)°.
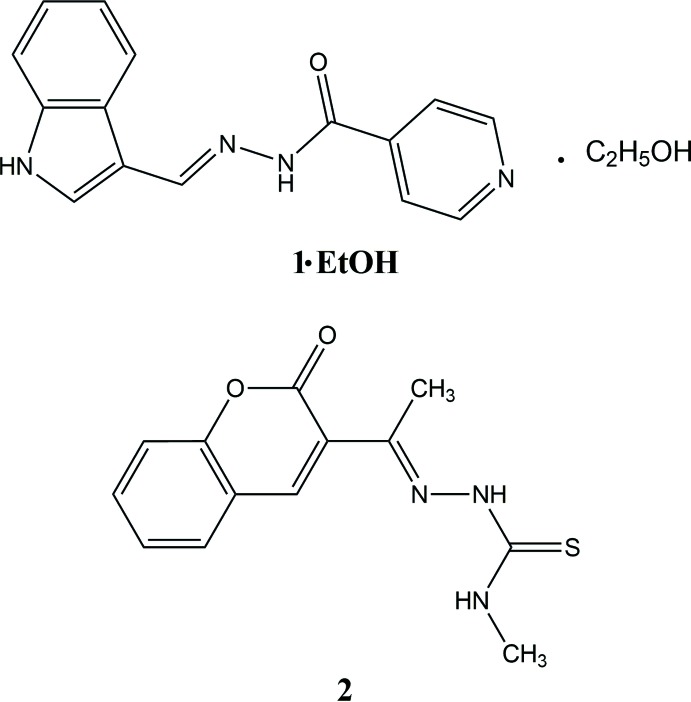


## Supra­molecular features   

**Figure 3:**
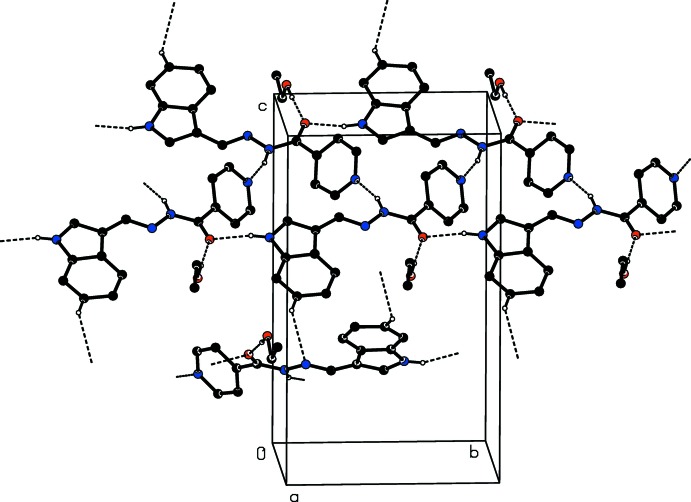
A packing diagram of compound **1·EtOH**, viewed along the *a* axis, showing the O—H⋯O, N—H⋯O, N—H⋯N and C—H⋯N inter­actions (dashed lines). For clarity, H atoms not involved in these inter­actions have been omitted.

The crystal packing of **1·EtOH** features O—H⋯O, N—H⋯O and N—H⋯N hydrogen bonds (Table 1 ▸), which link the mol­ecules into a tape structure running along the *b*-axis direction (Fig. 3 ▸). The tapes are weakly linked *via* a C—H⋯N inter­action (Table 1 ▸). In the N—H⋯O and N—H⋯N hydrogen bonds, atoms N1 and N3 act as donors to atoms O1 and N4, respectively, generating *C*(9) and *C*(7) chain motifs. The C—H⋯N inter­action generates a *C*(8) chain. Atom O1*S* of the ethanol mol­ecule acts as a donor in forming the O—H⋯O hydrogen bond with atom O1, which acts as a double acceptor.

**Figure 4:**
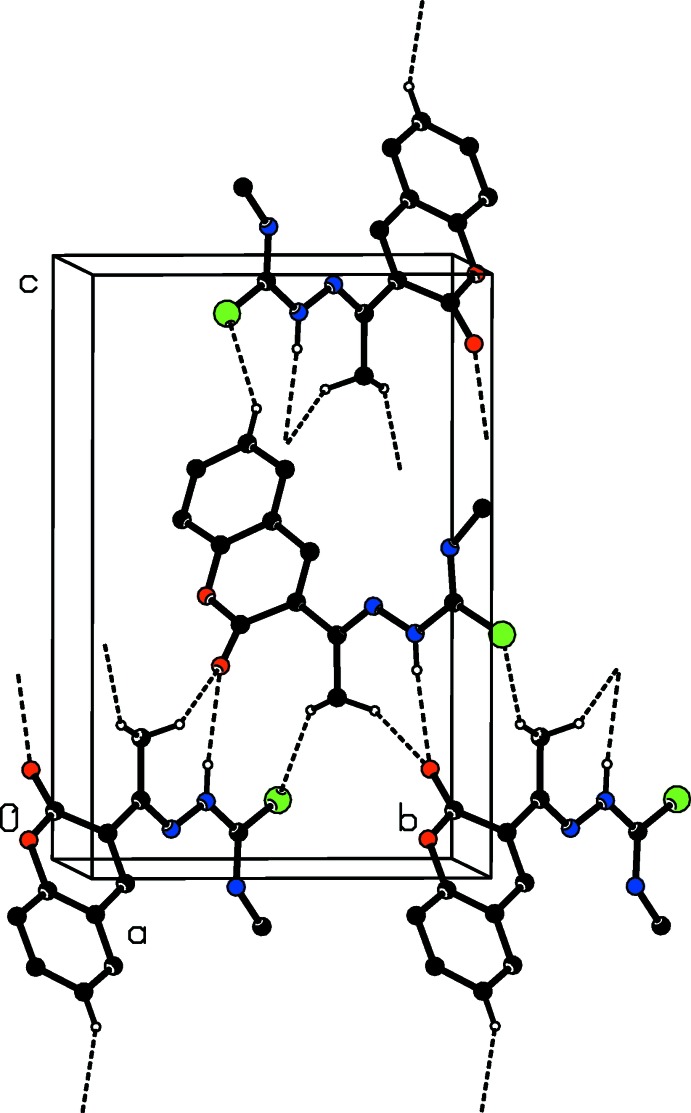
A crystal packing view of **2** along the *a* axis, showing the inter­molecular hydrogen-bonded network formed by N—H⋯O, C—H⋯O and C—H⋯S inter­actions (dashed lines). For clarity, H atoms not involved in these inter­actions have been omitted.

In **2**, the crystal packing features N—H⋯O, C—H⋯O and C—H⋯S inter­actions (Table 2 ▸). The mol­ecules are linked *via* N—H⋯O and C—H⋯O hydrogen bonds, forming a helical chain along the *b*-axis direction (Fig. 4 ▸). The chains are further linked *via* C—H⋯S inter­actions, forming a layer expanding parallel to (102). Atoms N2 and C11 act as donors to the double acceptor O2, generating *C*(7) and *C*(6) chains, respectively. As a result of these two hydrogen bonds, an 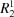
(7) ring motif is generated. In the C—H⋯S inter­actions, atoms C7 and C11 act as donors to the double acceptor S1, generating *C*(11) and *C*(7) chains, respectively.

## Database survey   

A search of the Cambridge Structural Database (Groom *et al.*, 2016 ▸) for the substructures **1** and **2** revealed several related Schiff base derivatives, including those with refcodes ADEKAW, ACIPIN, ADEZAL02 and APAQEP reported by Qiu *et al.* (2006 ▸), Lobana *et al.* (2012 ▸), Ilies *et al.* (2013 ▸) and Chainok *et al.* (2016 ▸), respectively.

## Synthesis and crystallization   

Compound **1** was synthesized by condensing equimolar amounts of 1*H*-indole-3-carbaldehyde (145 mg, 1 mmol) with nicotinic acid hydrazide (137 mg, 1 mmol) in ethanol. The reaction mixture was then refluxed on a water bath for 5 h and poured into crushed ice. The corresponding solid Schiff base that formed was filtered, washed several times with distilled water and dried under vacuum. The compound was recrystallized from an ethanol–chloro­form (1:3) solvent mixture, yielding the ethanol solvate compound, **1·EtOH**. Similarly, compound **2** was synthesized from equimolar amounts of 3-acetyl-2*H*-chromen-2-one (188 mg, 1 mmol) with *N*-methyl­hydrazine­carbo­thio­amide (105 mg, 1 mmol) in ethanol. Compound **2** was also recrystallized from an ethanol–chloro­form (1:3) solvent mixture.

## Refinement   

Crystal data, data collection and structure refinement details are summarized in Table 3 ▸. H atoms were refined as riding with N—H = 0.88, C—H = 0.95 or 0.98 Å and *U*_iso_(H) = 1.2 or 1.5*U*_eq_(parent atom). For **1·EtOH**, the methyl­ene H atoms of the ethanol solvent mol­ecule were refined independently under strong bond-length and angle restraints using *DFIX* to avoid a large residual electron-density peak near the methyl­ene C atom caused by the usual riding treatment of the H atoms. In **2**, *TWINABS* was employed to correct the data for absorption effects, as well as to separate hkl files for the domains with major and minor components; the twin ratio was observed to be 91:9. In the refinement, only the data of the major domain were used.

## Supplementary Material

Crystal structure: contains datablock(s) Global, 2, 1.EtOH. DOI: 10.1107/S205698901700411X/is5471sup1.cif

Structure factors: contains datablock(s) 1.EtOH. DOI: 10.1107/S205698901700411X/is54711.EtOHsup4.hkl

Structure factors: contains datablock(s) 2. DOI: 10.1107/S205698901700411X/is54712sup5.hkl

Supporting information file. DOI: 10.1107/S205698901700411X/is54711.EtOHsup4.cml

Supporting information file. DOI: 10.1107/S205698901700411X/is54712sup5.cml

CCDC references: 1537754, 1537753

Additional supporting information:  crystallographic information; 3D view; checkCIF report

## Figures and Tables

**Figure 2:**
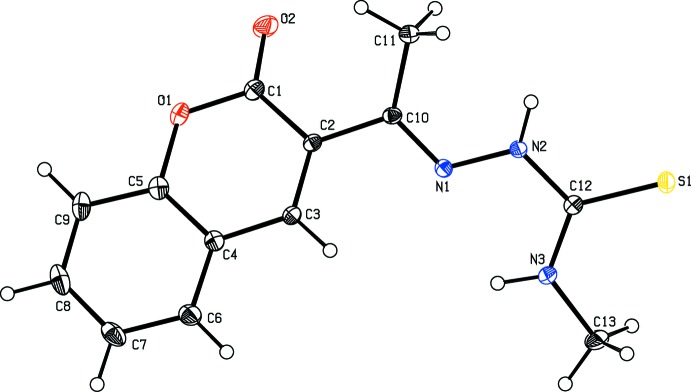
The mol­ecular structure of compound **2**, with the atom labelling. Displacement ellipsoids of non-H atoms are drawn at 30% probability level.

**Table 1:** Hydrogen-bond geometry (Å, °) for **1·EtOH** [Chem scheme1]

*D*—H⋯*A*	*D*—H	H⋯*A*	*D*⋯*A*	*D*—H⋯*A*
N1—H1⋯O1^i^	0.88	2.05	2.871 (3)	156
N3—H3⋯N4^ii^	0.88	2.14	2.979 (3)	159
C5—H5⋯N2^iii^	0.95	2.62	3.236 (3)	123
O1*S*—H1*S*⋯O1	0.84	1.90	2.742 (3)	177

Symmetry codes: (i) 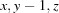; (ii) 
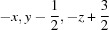
; (iii) 
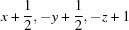
.

**Table 2:** Hydrogen-bond geometry (Å, °) for **2** [Chem scheme1]

*D*—H⋯*A*	*D*—H	H⋯*A*	*D*⋯*A*	*D*—H⋯*A*
N2—H2⋯O2^i^	0.88	2.39	3.269 (3)	175
C11—H11*A*⋯O2^i^	0.98	2.47	3.109 (3)	123
C7—H7⋯S1^ii^	0.95	2.85	3.711 (3)	151
C11—H11*B*⋯S1^iii^	0.98	2.87	3.728 (3)	146

Symmetry codes: (i) 
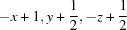
; (ii) 
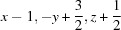
; (iii) 
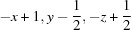
.

**Table 3:** Experimental details

	**1·EtOH**	**2**
Crystal data		
Chemical formula	C_15_H_12_N_4_O·C_2_H_6_O	C_13_H_13_N_3_O_2_S
*M*_r_	310.35	275.32
Crystal system, space group	Orthorhombic, *P*2_1_2_1_2_1_	Monoclinic, *P*2_1_/*c*
Temperature (K)	110	100
*a*, *b*, *c* (Å)	9.4692 (18), 9.9821 (19), 16.682 (3)	9.289 (4), 9.616 (4), 14.474 (6)
α, β, γ (°)	90, 90, 90	90, 90.825 (4), 90
*V* (Å^3^)	1576.9 (5)	1292.8 (9)
*Z*	4	4
Radiation type	Mo *K*α	Mo *K*α
μ (mm^−1^)	0.09	0.25
Crystal size (mm)	0.50 × 0.37 × 0.13	0.49 × 0.46 × 0.31

Data collection		
Diffractometer	Bruker APEXII CCD	Bruker APEXII CCD
Absorption correction	Multi-scan (*SADABS*; Bruker, 2008 ▸)	Multi-scan (*TWINABS*; Bruker, 2012 ▸)
*T*_min_, *T*_max_	0.618, 0.681	0.534, 0.746
No. of measured, independent and observed [*I* > 2σ(*I*)] reflections	39878, 3616, 3527	5480, 2902, 2285
*R*_int_	0.054	0.044
(sin θ/λ)_max_ (Å^−1^)	0.651	0.651

Refinement		
*R*[*F* ^2^ > 2σ(*F* ^2^)], *wR*(*F* ^2^), *S*	0.043, 0.119, 0.98	0.048, 0.116, 1.10
No. of reflections	3616	2902
No. of parameters	216	174
No. of restraints	3	0
H-atom treatment	H atoms treated by a mixture of independent and constrained refinement	H-atom parameters constrained
Δρ_max_, Δρ_min_ (e Å^−3^)	1.50, −0.36	0.30, −0.35
Absolute structure	Flack *x* determined using 1491 quotients [(*I* ^+^)−(*I* ^−^)]/[(*I* ^+^)+(*I* ^−^)] (Parsons *et al.*, 2013 ▸)	–
Absolute structure parameter	−0.2 (3)	–

Computer programs: *APEX2* and *SAINT* (Bruker, 2013 ▸), *SHELXS2014* and *SHELXS2013* (Sheldrick, 2008 ▸), *SHELXL2013* and *SHELXL2016* (Sheldrick, 2015 ▸) and *PLATON* (Spek, 2015 ▸).

## supplementary crystallographic information

### (1.EtOH) (E)-N'-[(1H-Indol-3-yl)methylidene]isonicotinohydrazide ethanol monosolvate Crystal data

**Table:**

C_15_H_12_N_4_O·C_2_H_6_O	*D*_x_ = 1.307 Mg m^−^^3^
*M**_r_* = 310.35	Mo *K*α radiation, λ = 0.71073 Å
Orthorhombic, *P*2_1_2_1_2_1_	Cell parameters from 9846 reflections
*a* = 9.4692 (18) Å	θ = 2.4–27.5°
*b* = 9.9821 (19) Å	µ = 0.09 mm^−^^1^
*c* = 16.682 (3) Å	*T* = 110 K
*V* = 1576.9 (5) Å^3^	Block, colorless
*Z* = 4	0.50 × 0.37 × 0.13 mm
*F*(000) = 656	

### (1.EtOH) (E)-N'-[(1H-Indol-3-yl)methylidene]isonicotinohydrazide ethanol monosolvate Data collection

**Table:**

Bruker APEXII CCD diffractometer	3527 reflections with *I* > 2σ(*I*)
φ and ω scans	*R*_int_ = 0.054
Absorption correction: multi-scan (SADABS; Bruker, 2008)	θ_max_ = 27.6°, θ_min_ = 2.4°
*T*_min_ = 0.618, *T*_max_ = 0.681	*h* = −12→12
39878 measured reflections	*k* = −12→12
3616 independent reflections	*l* = −21→21

### (1.EtOH) (E)-N'-[(1H-Indol-3-yl)methylidene]isonicotinohydrazide ethanol monosolvate Refinement

**Table:**

Refinement on *F*^2^	Hydrogen site location: mixed
Least-squares matrix: full	H atoms treated by a mixture of independent and constrained refinement
*R*[*F*^2^ > 2σ(*F*^2^)] = 0.043	*w* = 1/[σ^2^(*F*_o_^2^) + (0.077*P*)^2^ + 0.9574*P*] where *P* = (*F*_o_^2^ + 2*F*_c_^2^)/3
*wR*(*F*^2^) = 0.119	(Δ/σ)_max_ = 0.004
*S* = 0.98	Δρ_max_ = 1.50 e Å^−^^3^
3616 reflections	Δρ_min_ = −0.36 e Å^−^^3^
216 parameters	Absolute structure: Flack *x* determined using 1491 quotients [(*I*^+^)-(*I*^-^)]/[(*I*^+^)+(*I*^-^)] (Parsons *et al.*, 2013)
3 restraints	Absolute structure parameter: −0.2 (3)

### (1.EtOH) (E)-N'-[(1H-Indol-3-yl)methylidene]isonicotinohydrazide ethanol monosolvate Special details

**Table:**

Experimental. SADABS-2014/3 (Bruker, 2014) was used for absorption correction. wR2(int) was 0.1205 before and 0.0824 after correction. The Ratio of minimum to maximum transmission is 0.9082. The λ/2 correction factor is not present.
Geometry. All esds (except the esd in the dihedral angle between two l.s. planes) are estimated using the full covariance matrix. The cell esds are taken into account individually in the estimation of esds in distances, angles and torsion angles; correlations between esds in cell parameters are only used when they are defined by crystal symmetry. An approximate (isotropic) treatment of cell esds is used for estimating esds involving l.s. planes.
Refinement. 1. Fixed Uiso; at 1.2 times of: all C(H) groups, all N(H) groups and at 1.5 times of: C2S(H2SA, H2SB, H2SC) and O(H) groups 2. a. Aromatic/amide H refined with riding coordinates: N1(H1), N3(H3), C3(H3A), C4(H4), C5(H5), C6(H6), C7(H7), C9(H9), C12(H12), C13(H13), C14(H14), C15(H15) b. Idealised Me refined as rotating group: C11(H11A, H11B, H11C) 3. Strong restraints with DFIX were employed for methylene hydrogen atoms of the ethanol solvent molecule.

### (1.EtOH) (E)-N'-[(1H-Indol-3-yl)methylidene]isonicotinohydrazide ethanol monosolvate Fractional atomic coordinates and isotropic or equivalent isotropic displacement parameters (Å^2^)

**Table:**

	*x*	*y*	*z*	*U*_iso_*/*U*_eq_	
O1	0.41425 (19)	0.67417 (16)	0.63518 (11)	0.0184 (4)	
N1	0.4971 (2)	−0.05011 (19)	0.64904 (12)	0.0157 (4)	
H1	0.492857	−0.137698	0.654508	0.019*	
N2	0.3980 (2)	0.40446 (18)	0.66721 (11)	0.0140 (4)	
N3	0.2999 (2)	0.49844 (19)	0.69437 (12)	0.0137 (4)	
H3	0.228694	0.472947	0.724635	0.016*	
N4	−0.0322 (2)	0.8812 (2)	0.74337 (13)	0.0195 (4)	
C1	0.5943 (2)	0.0185 (2)	0.60295 (13)	0.0143 (4)	
C2	0.5644 (2)	0.1570 (2)	0.60882 (13)	0.0135 (4)	
C3	0.6449 (3)	0.2483 (2)	0.56436 (14)	0.0166 (5)	
H3A	0.625228	0.341561	0.566138	0.020*	
C4	0.7543 (3)	0.1987 (3)	0.51768 (15)	0.0201 (5)	
H4	0.809673	0.259218	0.486964	0.024*	
C5	0.7852 (3)	0.0605 (3)	0.51472 (15)	0.0204 (5)	
H5	0.862792	0.030250	0.483480	0.024*	
C6	0.7048 (3)	−0.0316 (2)	0.55637 (14)	0.0185 (5)	
H6	0.723861	−0.124959	0.553456	0.022*	
C7	0.4091 (2)	0.0393 (2)	0.68452 (14)	0.0156 (4)	
H7	0.333452	0.016491	0.719368	0.019*	
C8	0.4453 (2)	0.1688 (2)	0.66264 (13)	0.0140 (4)	
C9	0.3669 (2)	0.2839 (2)	0.68918 (13)	0.0142 (4)	
H9	0.289165	0.270747	0.724287	0.017*	
C10	0.3146 (2)	0.6275 (2)	0.67420 (13)	0.0133 (4)	
C11	0.1947 (2)	0.7155 (2)	0.70049 (14)	0.0139 (4)	
C12	0.1325 (2)	0.7050 (2)	0.77589 (14)	0.0156 (4)	
H12	0.165590	0.641316	0.813786	0.019*	
C13	0.0204 (2)	0.7902 (2)	0.79444 (14)	0.0185 (5)	
H13	−0.021080	0.783377	0.846143	0.022*	
C14	0.0301 (3)	0.8902 (2)	0.67103 (15)	0.0196 (5)	
H14	−0.005725	0.954149	0.634104	0.023*	
C15	0.1435 (2)	0.8117 (2)	0.64711 (15)	0.0168 (5)	
H15	0.185233	0.823122	0.595771	0.020*	
O1S	0.6541 (2)	0.6025 (2)	0.55370 (12)	0.0279 (4)	
H1S	0.582293	0.623623	0.580387	0.042*	
C1S	0.7704 (3)	0.5902 (2)	0.60520 (14)	0.0246 (5)	
H1SA	0.757 (3)	0.4957 (4)	0.6166 (9)	0.037*	
H1SB	0.776 (4)	0.6301 (9)	0.6581 (2)	0.037*	
C2S	0.9016 (3)	0.5715 (3)	0.5560 (2)	0.0314 (6)	
H2SA	0.889630	0.494186	0.520487	0.047*	
H2SB	0.982242	0.556124	0.591660	0.047*	
H2SC	0.918511	0.652049	0.523841	0.047*	

### (1.EtOH) (E)-N'-[(1H-Indol-3-yl)methylidene]isonicotinohydrazide ethanol monosolvate Atomic displacement parameters (Å^2^)

**Table:**

	*U*^11^	*U*^22^	*U*^33^	*U*^12^	*U*^13^	*U*^23^
O1	0.0179 (8)	0.0109 (7)	0.0262 (8)	−0.0002 (6)	0.0061 (7)	−0.0001 (6)
N1	0.0189 (9)	0.0099 (8)	0.0183 (9)	−0.0004 (7)	0.0013 (7)	0.0009 (7)
N2	0.0137 (8)	0.0113 (8)	0.0169 (9)	0.0022 (7)	0.0002 (7)	−0.0012 (7)
N3	0.0120 (8)	0.0116 (8)	0.0177 (9)	0.0004 (7)	0.0025 (7)	−0.0003 (7)
N4	0.0150 (9)	0.0153 (9)	0.0282 (10)	0.0019 (8)	0.0009 (8)	−0.0020 (8)
C1	0.0156 (10)	0.0125 (10)	0.0147 (9)	−0.0009 (8)	−0.0034 (8)	0.0008 (8)
C2	0.0138 (10)	0.0122 (10)	0.0143 (9)	0.0011 (7)	−0.0018 (8)	−0.0001 (8)
C3	0.0182 (11)	0.0137 (10)	0.0178 (10)	−0.0015 (8)	0.0003 (9)	−0.0003 (8)
C4	0.0207 (11)	0.0219 (12)	0.0177 (10)	−0.0021 (10)	0.0046 (9)	0.0005 (9)
C5	0.0181 (11)	0.0250 (13)	0.0180 (10)	0.0025 (9)	0.0018 (9)	−0.0022 (9)
C6	0.0213 (11)	0.0158 (10)	0.0184 (11)	0.0043 (9)	−0.0012 (9)	−0.0016 (9)
C7	0.0168 (10)	0.0130 (10)	0.0171 (10)	0.0004 (8)	−0.0004 (8)	0.0005 (8)
C8	0.0139 (10)	0.0129 (10)	0.0153 (9)	−0.0004 (8)	−0.0011 (8)	0.0005 (8)
C9	0.0133 (9)	0.0135 (10)	0.0157 (10)	0.0000 (8)	0.0002 (8)	0.0003 (8)
C10	0.0138 (10)	0.0118 (9)	0.0143 (10)	0.0014 (8)	−0.0011 (8)	−0.0020 (8)
C11	0.0126 (9)	0.0110 (9)	0.0181 (10)	−0.0012 (8)	−0.0006 (8)	−0.0029 (8)
C12	0.0151 (10)	0.0134 (10)	0.0183 (10)	0.0001 (8)	−0.0004 (8)	−0.0007 (8)
C13	0.0165 (10)	0.0186 (10)	0.0204 (11)	−0.0007 (9)	0.0032 (9)	−0.0021 (9)
C14	0.0180 (10)	0.0144 (10)	0.0263 (11)	0.0018 (9)	−0.0003 (9)	0.0028 (9)
C15	0.0162 (10)	0.0132 (10)	0.0212 (11)	−0.0007 (8)	0.0006 (9)	0.0011 (8)
O1S	0.0224 (9)	0.0324 (10)	0.0288 (10)	0.0047 (8)	0.0065 (7)	−0.0004 (8)
C1S	0.0269 (13)	0.0223 (12)	0.0246 (12)	−0.0020 (10)	0.0061 (10)	−0.0072 (10)
C2S	0.0208 (12)	0.0312 (14)	0.0423 (16)	−0.0029 (11)	0.0077 (12)	−0.0042 (12)

### (1.EtOH) (E)-N'-[(1H-Indol-3-yl)methylidene]isonicotinohydrazide ethanol monosolvate Geometric parameters (Å, º)

**Table:**

O1—C10	1.238 (3)	C7—C8	1.386 (3)
N1—C7	1.357 (3)	C7—H7	0.9500
N1—C1	1.381 (3)	C8—C9	1.437 (3)
N1—H1	0.8800	C9—H9	0.9500
N2—C9	1.292 (3)	C10—C11	1.501 (3)
N2—N3	1.396 (3)	C11—C12	1.393 (3)
N3—C10	1.339 (3)	C11—C15	1.396 (3)
N3—H3	0.8800	C12—C13	1.395 (3)
N4—C13	1.341 (3)	C12—H12	0.9500
N4—C14	1.346 (3)	C13—H13	0.9500
C1—C6	1.397 (3)	C14—C15	1.388 (3)
C1—C2	1.414 (3)	C14—H14	0.9500
C2—C3	1.401 (3)	C15—H15	0.9500
C2—C8	1.446 (3)	O1S—C1S	1.402 (3)
C3—C4	1.387 (3)	O1S—H1S	0.8400
C3—H3A	0.9500	C1S—C2S	1.500 (4)
C4—C5	1.411 (4)	C1S—H1SA	0.9700 (2)
C4—H4	0.9500	C1S—H1SB	0.9700 (2)
C5—C6	1.381 (3)	C2S—H2SA	0.9800
C5—H5	0.9500	C2S—H2SB	0.9800
C6—H6	0.9500	C2S—H2SC	0.9800
			
C7—N1—C1	109.00 (18)	N2—C9—H9	118.7
C7—N1—H1	125.5	C8—C9—H9	118.7
C1—N1—H1	125.5	O1—C10—N3	125.0 (2)
C9—N2—N3	112.49 (18)	O1—C10—C11	120.7 (2)
C10—N3—N2	119.74 (19)	N3—C10—C11	114.30 (19)
C10—N3—H3	120.1	C12—C11—C15	118.7 (2)
N2—N3—H3	120.1	C12—C11—C10	122.7 (2)
C13—N4—C14	116.9 (2)	C15—C11—C10	118.6 (2)
N1—C1—C6	129.1 (2)	C11—C12—C13	118.5 (2)
N1—C1—C2	108.2 (2)	C11—C12—H12	120.8
C6—C1—C2	122.6 (2)	C13—C12—H12	120.8
C3—C2—C1	119.3 (2)	N4—C13—C12	123.6 (2)
C3—C2—C8	134.4 (2)	N4—C13—H13	118.2
C1—C2—C8	106.20 (19)	C12—C13—H13	118.2
C4—C3—C2	118.1 (2)	N4—C14—C15	124.0 (2)
C4—C3—H3A	120.9	N4—C14—H14	118.0
C2—C3—H3A	120.9	C15—C14—H14	118.0
C3—C4—C5	121.6 (2)	C14—C15—C11	118.3 (2)
C3—C4—H4	119.2	C14—C15—H15	120.9
C5—C4—H4	119.2	C11—C15—H15	120.9
C6—C5—C4	121.3 (2)	C1S—O1S—H1S	109.5
C6—C5—H5	119.4	O1S—C1S—C2S	109.0 (2)
C4—C5—H5	119.4	O1S—C1S—H1SA	96.0 (14)
C5—C6—C1	117.0 (2)	C2S—C1S—H1SA	95.2 (13)
C5—C6—H6	121.5	O1S—C1S—H1SB	124.6 (19)
C1—C6—H6	121.5	C2S—C1S—H1SB	120 (2)
N1—C7—C8	110.3 (2)	H1SA—C1S—H1SB	103.2 (9)
N1—C7—H7	124.9	C1S—C2S—H2SA	109.5
C8—C7—H7	124.9	C1S—C2S—H2SB	109.5
C7—C8—C9	122.4 (2)	H2SA—C2S—H2SB	109.5
C7—C8—C2	106.3 (2)	C1S—C2S—H2SC	109.5
C9—C8—C2	131.2 (2)	H2SA—C2S—H2SC	109.5
N2—C9—C8	122.6 (2)	H2SB—C2S—H2SC	109.5
			
C9—N2—N3—C10	−177.7 (2)	C3—C2—C8—C9	0.1 (4)
C7—N1—C1—C6	179.6 (2)	C1—C2—C8—C9	−178.0 (2)
C7—N1—C1—C2	−1.0 (3)	N3—N2—C9—C8	174.45 (19)
N1—C1—C2—C3	−177.2 (2)	C7—C8—C9—N2	−178.1 (2)
C6—C1—C2—C3	2.2 (3)	C2—C8—C9—N2	−1.6 (4)
N1—C1—C2—C8	1.3 (2)	N2—N3—C10—O1	−3.5 (3)
C6—C1—C2—C8	−179.3 (2)	N2—N3—C10—C11	174.32 (18)
C1—C2—C3—C4	−1.8 (3)	O1—C10—C11—C12	−139.5 (2)
C8—C2—C3—C4	−179.8 (2)	N3—C10—C11—C12	42.5 (3)
C2—C3—C4—C5	−0.3 (4)	O1—C10—C11—C15	40.6 (3)
C3—C4—C5—C6	2.0 (4)	N3—C10—C11—C15	−137.4 (2)
C4—C5—C6—C1	−1.6 (4)	C15—C11—C12—C13	0.7 (3)
N1—C1—C6—C5	178.8 (2)	C10—C11—C12—C13	−179.2 (2)
C2—C1—C6—C5	−0.5 (3)	C14—N4—C13—C12	−1.1 (3)
C1—N1—C7—C8	0.2 (3)	C11—C12—C13—N4	0.7 (4)
N1—C7—C8—C9	177.8 (2)	C13—N4—C14—C15	0.0 (4)
N1—C7—C8—C2	0.6 (3)	N4—C14—C15—C11	1.4 (4)
C3—C2—C8—C7	177.1 (2)	C12—C11—C15—C14	−1.7 (3)
C1—C2—C8—C7	−1.1 (2)	C10—C11—C15—C14	178.2 (2)

### (1.EtOH) (E)-N'-[(1H-Indol-3-yl)methylidene]isonicotinohydrazide ethanol monosolvate Hydrogen-bond geometry (Å, º)

**Table:**

*D*—H···*A*	*D*—H	H···*A*	*D*···*A*	*D*—H···*A*
N1—H1···O1^i^	0.88	2.05	2.871 (3)	156
N3—H3···N4^ii^	0.88	2.14	2.979 (3)	159
C5—H5···N2^iii^	0.95	2.62	3.236 (3)	123
O1*S*—H1*S*···O1	0.84	1.90	2.742 (3)	177

Symmetry codes: (i) *x*, *y*−1, *z*; (ii) −*x*, *y*−1/2, −*z*+3/2; (iii) *x*+1/2, −*y*+1/2, −*z*+1.

### (2) (E)-N'-Methyl-2-[1-(2-oxo-2H-chromen-3-yl)ethylidene]hydrazinecarbothioamide Crystal data

**Table:**

C_13_H_13_N_3_O_2_S	*F*(000) = 576
*M**_r_* = 275.32	*D*_x_ = 1.415 Mg m^−^^3^
Monoclinic, *P*2_1_/*c*	Mo *K*α radiation, λ = 0.71073 Å
*a* = 9.289 (4) Å	Cell parameters from 4293 reflections
*b* = 9.616 (4) Å	θ = 2.2–27.3°
*c* = 14.474 (6) Å	µ = 0.25 mm^−^^1^
β = 90.825 (4)°	*T* = 100 K
*V* = 1292.8 (9) Å^3^	Block, yellow
*Z* = 4	0.49 × 0.46 × 0.31 mm

### (2) (E)-N'-Methyl-2-[1-(2-oxo-2H-chromen-3-yl)ethylidene]hydrazinecarbothioamide Data collection

**Table:**

Bruker APEXII CCD diffractometer	2285 reflections with *I* > 2σ(*I*)
φ and ω scans	*R*_int_ = 0.044
Absorption correction: multi-scan (*TWINABS*; Bruker, 2012)	θ_max_ = 27.6°, θ_min_ = 2.2°
*T*_min_ = 0.534, *T*_max_ = 0.746	*h* = −12→12
5480 measured reflections	*k* = −12→12
2902 independent reflections	*l* = 0→18

### (2) (E)-N'-Methyl-2-[1-(2-oxo-2H-chromen-3-yl)ethylidene]hydrazinecarbothioamide Refinement

**Table:**

Refinement on *F*^2^	0 restraints
Least-squares matrix: full	Hydrogen site location: inferred from neighbouring sites
*R*[*F*^2^ > 2σ(*F*^2^)] = 0.048	H-atom parameters constrained
*wR*(*F*^2^) = 0.116	*w* = 1/[σ^2^(*F*_o_^2^) + (0.0375*P*)^2^ + 0.711*P*] where *P* = (*F*_o_^2^ + 2*F*_c_^2^)/3
*S* = 1.10	(Δ/σ)_max_ = 0.001
2902 reflections	Δρ_max_ = 0.30 e Å^−^^3^
174 parameters	Δρ_min_ = −0.35 e Å^−^^3^

### (2) (E)-N'-Methyl-2-[1-(2-oxo-2H-chromen-3-yl)ethylidene]hydrazinecarbothioamide Special details

**Table:**

Experimental. For component 1: wR2(int) was 0.1337 before and 0.0605 after correction. The ratio of minimum to maximum transmission is 0.72. The λ/2 correction factor is not presentFinal HKLF 4 output contains 20988 reflections, Rint = 0.0871 (9738 with I > 3sig(I), Rint = 0.0747)
Geometry. All esds (except the esd in the dihedral angle between two l.s. planes) are estimated using the full covariance matrix. The cell esds are taken into account individually in the estimation of esds in distances, angles and torsion angles; correlations between esds in cell parameters are only used when they are defined by crystal symmetry. An approximate (isotropic) treatment of cell esds is used for estimating esds involving l.s. planes.
Refinement. The absorption correction program TWINABS2 was employed to correct the data for absorption effects, as well as to separate hkl files for the domains with major component, which was used for further analysis.1. Fixed Uiso; at 1.2 times of: All C(H) groups, all N(H) groups at 1.5 times of: all C(H, H, H) groups 2. a. Aromatic/amide H refined with riding coordinates: N2(H2), N3(H3), C3(H3A), C6(H6), C7(H7), C8(H8), C9(H9) b. Idealised Me refined as rotating group: C11(H11A, H11B, H11C), C13(H13A, H13B, H13C)

### (2) (E)-N'-Methyl-2-[1-(2-oxo-2H-chromen-3-yl)ethylidene]hydrazinecarbothioamide Fractional atomic coordinates and isotropic or equivalent isotropic displacement parameters (Å^2^)

**Table:**

	*x*	*y*	*z*	*U*_iso_*/*U*_eq_	
S1	0.80937 (6)	1.04558 (5)	0.39682 (4)	0.02114 (16)	
O1	0.21617 (15)	0.36330 (14)	0.44249 (10)	0.0215 (3)	
O2	0.37162 (18)	0.38370 (16)	0.33164 (11)	0.0314 (4)	
N1	0.52878 (17)	0.75100 (17)	0.43518 (11)	0.0160 (4)	
N2	0.61381 (18)	0.84670 (17)	0.39257 (11)	0.0173 (4)	
H2	0.6171	0.8510	0.3319	0.021*	
N3	0.67181 (19)	0.92800 (18)	0.53607 (11)	0.0197 (4)	
H3	0.6007	0.8755	0.5554	0.024*	
C1	0.3192 (2)	0.4382 (2)	0.39822 (14)	0.0200 (4)	
C2	0.3575 (2)	0.5745 (2)	0.43650 (13)	0.0158 (4)	
C3	0.3017 (2)	0.6138 (2)	0.51797 (13)	0.0164 (4)	
H3A	0.3296	0.7005	0.5441	0.020*	
C4	0.2019 (2)	0.5287 (2)	0.56577 (13)	0.0182 (4)	
C5	0.1581 (2)	0.4043 (2)	0.52496 (14)	0.0193 (4)	
C6	0.1375 (2)	0.5677 (2)	0.64904 (14)	0.0256 (5)	
H6	0.1641	0.6528	0.6780	0.031*	
C7	0.0363 (2)	0.4834 (3)	0.68876 (15)	0.0311 (6)	
H7	−0.0071	0.5099	0.7452	0.037*	
C8	−0.0026 (2)	0.3591 (3)	0.64601 (16)	0.0310 (6)	
H8	−0.0720	0.3010	0.6743	0.037*	
C9	0.0567 (2)	0.3179 (2)	0.56388 (15)	0.0256 (5)	
H9	0.0289	0.2332	0.5349	0.031*	
C10	0.4546 (2)	0.6670 (2)	0.38477 (13)	0.0164 (4)	
C11	0.4552 (3)	0.6675 (3)	0.28152 (14)	0.0309 (6)	
H11A	0.4431	0.7630	0.2590	0.046*	
H11B	0.3760	0.6097	0.2579	0.046*	
H11C	0.5470	0.6302	0.2599	0.046*	
C12	0.6937 (2)	0.9357 (2)	0.44612 (13)	0.0159 (4)	
C13	0.7581 (2)	1.0010 (2)	0.60437 (14)	0.0273 (5)	
H13A	0.8603	0.9825	0.5938	0.041*	
H13B	0.7326	0.9689	0.6663	0.041*	
H13C	0.7399	1.1011	0.5994	0.041*	

### (2) (E)-N'-Methyl-2-[1-(2-oxo-2H-chromen-3-yl)ethylidene]hydrazinecarbothioamide Atomic displacement parameters (Å^2^)

**Table:**

	*U*^11^	*U*^22^	*U*^33^	*U*^12^	*U*^13^	*U*^23^
S1	0.0185 (3)	0.0218 (3)	0.0231 (3)	−0.0043 (2)	0.0011 (2)	0.0012 (2)
O1	0.0192 (8)	0.0158 (7)	0.0295 (8)	−0.0012 (6)	−0.0026 (6)	−0.0015 (6)
O2	0.0320 (9)	0.0280 (9)	0.0343 (9)	−0.0001 (7)	0.0055 (7)	−0.0140 (7)
N1	0.0141 (8)	0.0183 (9)	0.0155 (8)	0.0000 (7)	−0.0004 (6)	0.0013 (6)
N2	0.0162 (8)	0.0225 (9)	0.0130 (8)	−0.0038 (7)	−0.0018 (6)	0.0010 (7)
N3	0.0206 (9)	0.0219 (9)	0.0164 (9)	−0.0043 (7)	−0.0021 (7)	−0.0015 (7)
C1	0.0168 (10)	0.0206 (11)	0.0226 (11)	0.0015 (9)	−0.0033 (8)	−0.0010 (9)
C2	0.0133 (9)	0.0178 (10)	0.0161 (10)	0.0009 (8)	−0.0036 (7)	−0.0009 (8)
C3	0.0138 (10)	0.0181 (10)	0.0173 (10)	0.0009 (8)	−0.0036 (7)	0.0001 (8)
C4	0.0138 (10)	0.0235 (11)	0.0171 (10)	0.0018 (8)	−0.0053 (7)	0.0054 (8)
C5	0.0140 (10)	0.0200 (10)	0.0237 (11)	0.0045 (8)	−0.0051 (8)	0.0066 (8)
C6	0.0212 (11)	0.0361 (13)	0.0195 (11)	−0.0010 (10)	−0.0027 (8)	0.0017 (9)
C7	0.0218 (12)	0.0522 (16)	0.0193 (11)	−0.0009 (11)	−0.0017 (9)	0.0119 (10)
C8	0.0171 (11)	0.0420 (14)	0.0339 (13)	−0.0039 (10)	−0.0045 (9)	0.0221 (11)
C9	0.0172 (11)	0.0237 (11)	0.0356 (13)	−0.0026 (9)	−0.0070 (9)	0.0114 (9)
C10	0.0149 (10)	0.0193 (10)	0.0151 (10)	0.0015 (8)	−0.0009 (7)	−0.0019 (8)
C11	0.0347 (14)	0.0419 (14)	0.0162 (11)	−0.0165 (11)	0.0014 (9)	−0.0042 (10)
C12	0.0131 (9)	0.0162 (10)	0.0184 (10)	0.0035 (8)	−0.0027 (7)	0.0008 (8)
C13	0.0269 (12)	0.0347 (13)	0.0202 (11)	−0.0033 (11)	−0.0069 (9)	−0.0041 (9)

### (2) (E)-N'-Methyl-2-[1-(2-oxo-2H-chromen-3-yl)ethylidene]hydrazinecarbothioamide Geometric parameters (Å, º)

**Table:**

S1—C12	1.674 (2)	C4—C6	1.404 (3)
O1—C1	1.365 (2)	C5—C9	1.382 (3)
O1—C5	1.375 (3)	C6—C7	1.374 (3)
O2—C1	1.206 (2)	C6—H6	0.9500
N1—C10	1.282 (3)	C7—C8	1.392 (4)
N1—N2	1.366 (2)	C7—H7	0.9500
N2—C12	1.367 (3)	C8—C9	1.375 (3)
N2—H2	0.8800	C8—H8	0.9500
N3—C12	1.323 (3)	C9—H9	0.9500
N3—C13	1.446 (3)	C10—C11	1.494 (3)
N3—H3	0.8800	C11—H11A	0.9800
C1—C2	1.464 (3)	C11—H11B	0.9800
C2—C3	1.349 (3)	C11—H11C	0.9800
C2—C10	1.479 (3)	C13—H13A	0.9800
C3—C4	1.423 (3)	C13—H13B	0.9800
C3—H3A	0.9500	C13—H13C	0.9800
C4—C5	1.392 (3)		
			
C1—O1—C5	122.86 (16)	C6—C7—H7	120.1
C10—N1—N2	118.48 (16)	C8—C7—H7	120.1
N1—N2—C12	118.61 (16)	C9—C8—C7	121.8 (2)
N1—N2—H2	120.7	C9—C8—H8	119.1
C12—N2—H2	120.7	C7—C8—H8	119.1
C12—N3—C13	123.65 (18)	C8—C9—C5	117.6 (2)
C12—N3—H3	118.2	C8—C9—H9	121.2
C13—N3—H3	118.2	C5—C9—H9	121.2
O2—C1—O1	116.07 (18)	N1—C10—C2	114.68 (17)
O2—C1—C2	126.37 (19)	N1—C10—C11	123.86 (18)
O1—C1—C2	117.55 (17)	C2—C10—C11	121.29 (17)
C3—C2—C1	119.16 (18)	C10—C11—H11A	109.5
C3—C2—C10	121.28 (18)	C10—C11—H11B	109.5
C1—C2—C10	119.56 (17)	H11A—C11—H11B	109.5
C2—C3—C4	121.67 (18)	C10—C11—H11C	109.5
C2—C3—H3A	119.2	H11A—C11—H11C	109.5
C4—C3—H3A	119.2	H11B—C11—H11C	109.5
C5—C4—C6	117.92 (19)	N3—C12—N2	115.66 (17)
C5—C4—C3	118.43 (18)	N3—C12—S1	124.32 (15)
C6—C4—C3	123.51 (19)	N2—C12—S1	120.02 (15)
O1—C5—C9	117.38 (19)	N3—C13—H13A	109.5
O1—C5—C4	119.94 (18)	N3—C13—H13B	109.5
C9—C5—C4	122.7 (2)	H13A—C13—H13B	109.5
C7—C6—C4	120.3 (2)	N3—C13—H13C	109.5
C7—C6—H6	119.9	H13A—C13—H13C	109.5
C4—C6—H6	119.9	H13B—C13—H13C	109.5
C6—C7—C8	119.7 (2)		
			
C10—N1—N2—C12	−179.56 (18)	C5—C4—C6—C7	1.0 (3)
C5—O1—C1—O2	172.55 (18)	C3—C4—C6—C7	176.49 (19)
C5—O1—C1—C2	−6.3 (3)	C4—C6—C7—C8	−0.1 (3)
O2—C1—C2—C3	−171.9 (2)	C6—C7—C8—C9	−0.7 (3)
O1—C1—C2—C3	6.9 (3)	C7—C8—C9—C5	0.5 (3)
O2—C1—C2—C10	8.9 (3)	O1—C5—C9—C8	−179.46 (18)
O1—C1—C2—C10	−172.34 (17)	C4—C5—C9—C8	0.4 (3)
C1—C2—C3—C4	−2.7 (3)	N2—N1—C10—C2	−175.51 (16)
C10—C2—C3—C4	176.51 (17)	N2—N1—C10—C11	−0.1 (3)
C2—C3—C4—C5	−2.2 (3)	C3—C2—C10—N1	28.8 (3)
C2—C3—C4—C6	−177.73 (19)	C1—C2—C10—N1	−152.03 (18)
C1—O1—C5—C9	−178.67 (18)	C3—C2—C10—C11	−146.7 (2)
C1—O1—C5—C4	1.5 (3)	C1—C2—C10—C11	32.4 (3)
C6—C4—C5—O1	178.71 (18)	C13—N3—C12—N2	172.14 (19)
C3—C4—C5—O1	3.0 (3)	C13—N3—C12—S1	−8.2 (3)
C6—C4—C5—C9	−1.1 (3)	N1—N2—C12—N3	−5.4 (3)
C3—C4—C5—C9	−176.87 (18)	N1—N2—C12—S1	174.97 (13)

### (2) (E)-N'-Methyl-2-[1-(2-oxo-2H-chromen-3-yl)ethylidene]hydrazinecarbothioamide Hydrogen-bond geometry (Å, º)

**Table:**

*D*—H···*A*	*D*—H	H···*A*	*D*···*A*	*D*—H···*A*
N2—H2···O2^i^	0.88	2.39	3.269 (3)	175
C11—H11*A*···O2^i^	0.98	2.47	3.109 (3)	123
C7—H7···S1^ii^	0.95	2.85	3.711 (3)	151
C11—H11*B*···S1^iii^	0.98	2.87	3.728 (3)	146

Symmetry codes: (i) −*x*+1, *y*+1/2, −*z*+1/2; (ii) *x*−1, −*y*+3/2, *z*+1/2; (iii) −*x*+1, *y*−1/2, −*z*+1/2.
